# Retrograde vacuum-assisted MiniPCNL system for large distal ureteric calculus removal: A case report

**DOI:** 10.1016/j.eucr.2023.102622

**Published:** 2023-11-16

**Authors:** Tarapon Setthawong, Theerawech Namwongsa, Chinnakhet Ketsuwan

**Affiliations:** Division of Urology, Department of Surgery, Faculty of Medicine Ramathibodi Hospital, Mahidol University, Bangkok, Thailand

**Keywords:** Distal ureteric calculus, MiniPCNL, Vacuum-assisted extraction

## Abstract

Large ureteral calculi are commonly associated with severe colic pain, complex urinary tract infections, severe hematuria, hydronephrosis, and renal deterioration, often requiring immediate surgical intervention. Ureteroscopy is a favored treatment due to its higher stone-free rates; however, it encounters difficulties in cases of a high burden of distal ureteral stones. We present a case where a patient with a significant ureteral calculus was effectively treated with a vacuum-assisted mini-percutaneous nephrolithotomy system in retrograde approach. This intervention enabled the complete removal of the stone, leading to the patient's full recovery without complications.

## 1 Introduction

Urolithiasis, characterized by a prevalence of 1 %–5 % in Asia and 7 %–13 % in North America, ranks as a highly prevalent urological disease worldwide. Twenty percent of lithiasis cases are ureteral, with 68 % occurring in the distal part. Large ureteral calculi typically result in intense colic pain, complicated urinary tract infections, gross hematuria, hydronephrosis, and renal deterioration, necessitating prompt surgical intervention. The European Association of Urology Guidelines recommend extracorporeal shockwave lithotripsy (ESWL) and ureteroscopy (URS) for treating ureteric calculi.^1^^,^^2^ However, ESWL necessitates multiple sessions and thorough follow-ups for full clearance, limiting its efficacy for larger stones. Conversely, URS offers superior stone-free rates (SFR) but faces challenges with a heavy burden of distal ureteral stones, potentially leading to prolonged procedures and increased septicemia risk. This scenario accentuates the critical need for innovating new, efficacious strategies tailored for the treatment of large distal ureteric calculi, aiming at enhancing safety and procedural efficiency while optimizing patient outcomes.

To our knowledge, we are documenting an inaugural clinical case that employs a miniature nephroscope alongside a percutaneous nephrolithotomy (PCNL) vacuum-assisted system for a patient with a significant ureteric calculus.

## 2 Case report

**Fig. 1:**
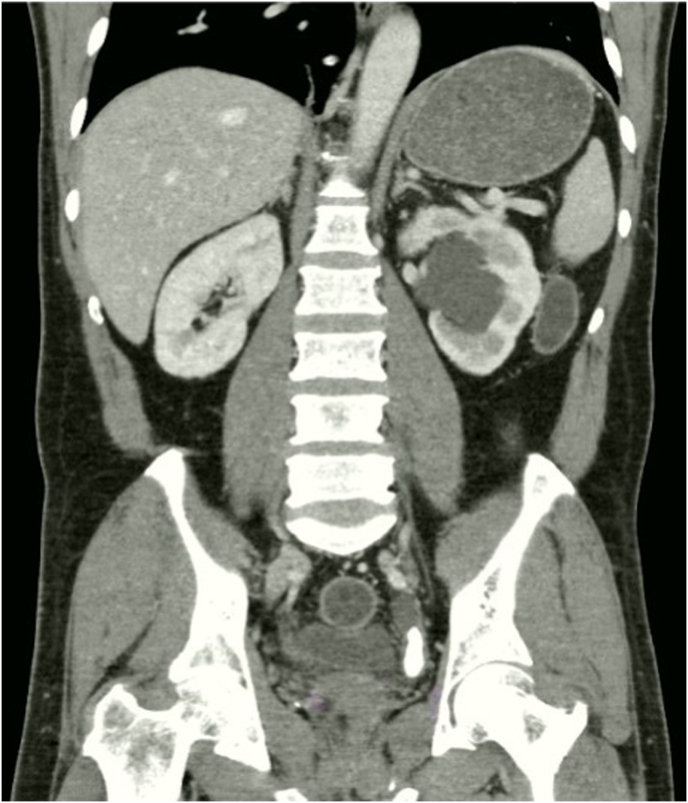
A Computerized tomography scan illustrating a left distal ureteric calculus.

A 58-year-old male with colonic cancer under oncology surveillance had a colon scan that accidently discovered a large left distal ureteric stone without any symptoms. His blood creatinine level was 1.0 mg/dL. A computed tomography scan showed a 10 × 11 × 28 mm^3^ calculus with a 750 Hounsfield unit density in the left distal ureter (Fig. 1). Excretion in the left kidney was moderately reduced, while normal function was observed in the right kidney. To preserve the left kidney function during a lengthy wait until the operation, a preoperative 6Fr double J stent was inserted into this patient. After evaluating multiple treatment options with the patient, we determined that utilizing a miniature nephroscope and vacuum-assisted MiniPCNL system in the lithotomy position would be optimal.

**Fig. 2:**
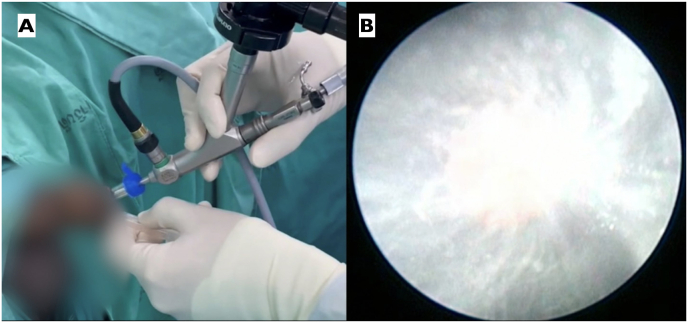
A, Retrograde vacuum-assisted MiniPCNL for ureteral calculus fragmentation; B, Extraction of ureteral stone powder and stone fragments via suction technique.

**Fig. 3:**
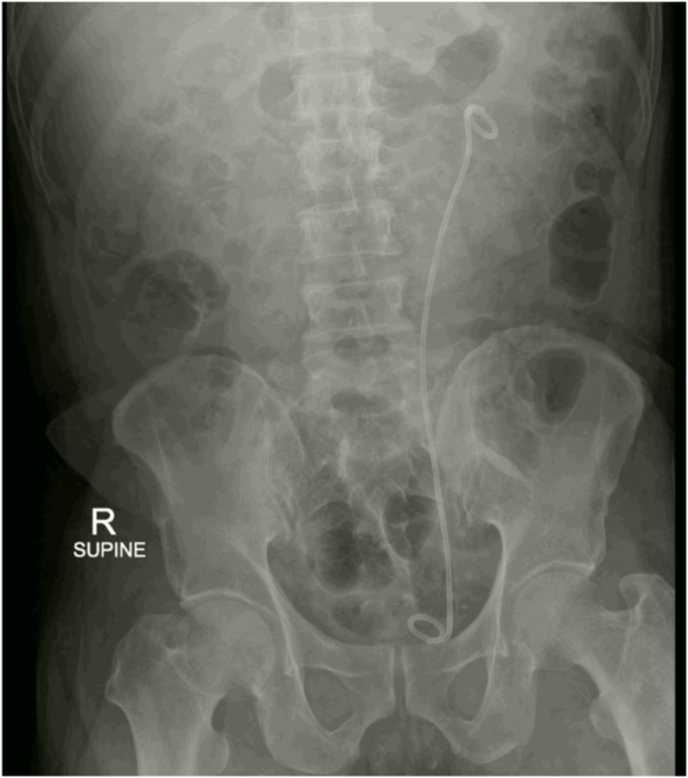
Postoperative abdominal radiography.

During the induction phase of general anesthesia, prophylactic intravenous cefuroxime was administered, and the patient was positioned in the lithotomy position. The existing DJ stent was removed, and a 5Fr ureteral catheter was cystoscopically inserted into the left ureter, delineating the collecting system with contrast dye. A hybrid guidewire (Sensor™, Boston Scientific, Natick, MA) was inserted into the ureteric orifice, followed by the placement of a 14/16 Fr Clear Petra disposable nephrostomic sheath (Well Lead Medical Co.) assembled with its stylet tip positioned below the distal part of the calculus under fluoroscopic guidance. The UROMAT E.A.S.I. pressure-controlled double-roller pump (Karl Storz, Tuttlingen, Germany) was utilized, maintaining a low pressure at 20 mmHg. A 12Fr miniature nephroscope MIP-M system (Karl Storz, Tuttlingen, Germany) was introduced via the sheath (Fig. 2A). Laser lithotripsy was executed using a fragmentation technique with a 120 W Ho:YAG laser (Lumenis, San Jose, CA) and a 550 μm core laser fiber operating at an energy of 1.0 J and a rate of 10 Hz. Stone powder, stone fragments, and irrigation fluid were continuously aspirated using a suction system (Fig. 2B). A retrograde 6Fr double-J stent was placed at the procedure's conclusion. The total operative time was 50 minutes, with 12 minutes dedicated to laser usage. Postoperative abdominal plain films confirmed the absence of residual stones and the appropriate placement of the double-J stent (Fig. 3). The patient's postoperative recovery was uneventful. The stent was cystoscopically removed 3 weeks post-discharge. Stone composition analysis was not conducted due to financial constraints.

## 3 Discussion

Urolithiasis remains a prevalent global health issue, with incidence rates on the rise. Endourologic techniques are typically favored in the surgical management of this condition due to their minimally invasive nature.^3^ Ureteroscopic lithotripsy is routinely employed for large ureteric and renal stones up to 2 cm in size. However, this modality is not without its limitations. Challenges include the retrieval of rough stone fragments, constrained visual fields, extended operative durations, and elevated intrarenal pressures. These factors necessitate ongoing advancements to enhance the efficacy and safety of urolithiasis management procedures.

Since Hugh Hampton Young's inception of the rigid ureteroscopic procedure in 1912 and Bagley's introduction of flexible ureteroscopy in 1983, the foundational technique of utilizing normal saline for irrigation remains constant. This practice enhances stone clearance and intraoperative visualization and mitigates thermal effects on tissues. Nonetheless, excessive pressure settings, because of the high perfusion rate of normal saline, can precipitate adverse outcomes. Significant data from animal studies illustrate this concern. Kidney injuries and reduced arterial blood flow have been documented at intrarenal pressures of 20–40 cm H_2_O. Pyelovenous backflow is identified at 40.8–47.6 cm H_2_O, while forniceal rupture risks escalate at pressures spanning 81.6–95.2 cm H_2_O. These complications can facilitate the systemic absorption of bacteria and their toxins, subsequently elevating the risk of life-threatening sepsis post-ureteroscopy, with incidence rates ranging from 0 to 4.5 %.^4^ Therefore, meticulous monitoring and management of saline pressure settings are paramount to circumventing these hazardous implications, underscoring the imperative for refined protocols and innovations in this domain.

In the past quarter of a century, not only have surgical instruments, artificial intelligence, and virtual reality innovations improved, but the techniques of endourological procedures have also been adjusted.^5^, ^6^, ^7^ Suction techniques have been an integral component during PCNL, often coupled with ultrasound and ballistic devices, for effective stone gravel evacuation. However, their incorporation in treating lower distal ureter stones has been conspicuously absent.^8^ To the best of our knowledge, our report presents the first case that integrates multiple innovative procedures for distal ureteric calculi treatment. The incorporation of a suction system is instrumental in maintaining intrarenal pressure at modest 20 mmHg. This pressure modulation not only attenuates postoperative complications but also augments visibility and expedites the procedural timeline. Furthermore, the employment of a miniature nephroscope in the retrograde approach is highlighted, boasting a substantial working channel that facilitates enhanced irrigation fluid flow and accommodates a larger laser fiber (550 μm) optimized for rapid stone fragmentation. These advancements mark a pivotal transition in the landscape of endourological interventions, promising enhanced efficacy and safety. However, this approach is only advisable when dealing with a wide ureter, as there is a risk of ureteral damage and stricture formation. In cases where the ureter's capacity is a concern, we recommend the preoperative placement of a DJ stent to mitigate these risks.

## 4 Conclusion

We successfully treated a very large distal ureteral calculus using a vacuum-assisted MiniPCNL system in the retrograde route. This integrative approach is designed to achieve comprehensive stone clearance, decrease operative duration, and mitigate associated complications.

## Declaration of competing interest

None.
